# Modelling growth curves of the normal infant’s mandible: 3D measurements using computed tomography

**DOI:** 10.1007/s00784-021-03937-1

**Published:** 2021-04-16

**Authors:** Jan Aart M. Schipper, Manouk J. S. van Lieshout, Stefan Böhringer, Bonnie L. Padwa, Simon G. F. Robben, Rick R. van Rijn, Maarten J. Koudstaal, Maarten H. Lequin, Eppo B. Wolvius

**Affiliations:** 1 Department of Oral and Maxillofacial Surgery, Erasmus University Medical Centre, Sophia’s Children’s Hospital, ‘s Gravendijkwal 230, 3015 CE Rotterdam, The Netherlands; 2 Department of Biomedical Data Sciences, Leiden University Medical Centre, Leiden, The Netherlands; 3 Department of Plastic and Oral Surgery, Boston Children’s Hospital, Boston, MA USA; 4 Department of Radiology, Maastricht University Medical Centre, Maastricht, The Netherlands; 5 Pediatric Radiology, Emma Children’s Hospital/Academic Medical Centre, Amsterdam, The Netherlands; 6 Department of Radiology, Wilhelmina Children’s Hospital, University Medical Centre Utrecht, Utrecht University, Utrecht, The Netherlands

**Keywords:** Mandible, Normal, Infant, Growth, Three-dimensional, Micrognathia

## Abstract

**Objectives:**

Data on normal mandibular development in the infant is lacking though essential to understand normal growth patterns and to discriminate abnormal growth. The aim of this study was to provide normal linear measurements of the mandible using computed tomography performed in infants from 0 to 2 years of age.

**Material and methods:**

3D voxel software was used to calculate mandibular body length, mandibular ramus length, bicondylar width, bigonial width and the gonial angle. Intra- and inter-rater reliability was assessed for these measurements. They were found to be sufficient for all distances; intra-class correlation coefficients were all above 0.9. Regression analysis for growth modelling was performed.

**Results:**

In this multi-centre retrospective study, 109 CT scans were found eligible that were performed for various reasons (e.g. trauma, craniosynostosis, craniofacial abscesses). Craniosynostosis patients had larger mandibular measurements compared to non-craniosynostosis patients and were therefore excluded. Fifty-one CT scans were analysed.

**Conclusions:**

Analysis showed that the mandible increases more in size vertically (the mandibular ramus) than horizontally (the mandibular body). Most of the mandibular growth occurs in the first 6 months.

**Clinical relevance:**

These growth models provide insight into normal mandibular development in the first 2 years of life. This reference data facilitates discrimination between normal and abnormal mandibular growth.

**Supplementary Information:**

The online version contains supplementary material available at 10.1007/s00784-021-03937-1.

## Introduction

The mandible is a common site of congenital abnormality. Neonatal micrognathia has an incidence of approximately 1:500–1600 births [1–3]. Foetal micrognathia may lead to severe functional problems shortly after birth. Besides upper airway problems, also feeding, swallowing and, later in life, speech problems may necessitate a multidisciplinary approach. In case of severe upper airway problems, some physicians rely on a physiologic intrinsic growth of the mandible in the first 2 years of life and tend to treat the infants non-surgically whenever possible. Others advocate a more aggressive approach with interventions like mandibular distraction and advocate to operate on these patients very early in life.

Data on normal mandibular development is essential to evaluate and to recognize abnormal mandibular size and growth. However, there are only a few studies documenting mandibular development in early life, and these show that the most rapid mandibular growth occurs during the first year of life [4, 5]. Several prenatal and postnatal mandibular measurements have been obtained. Prenatally, mandibular hypoplasia can be objectively diagnosed using the inferior facial angle, jaw index or antero-posterior diameter with ultrasound examinations or magnetic resonance imaging (MRI) measurements [1, 6–8]. Postnatally, several techniques can be used to assess the size and growth of the mandible: measurements from direct anthropometry with the use of callipers, two-dimensional (2D) cephalometry, stereophotogrammetry, MRI or CT. A complicating factor in young infants is that they cannot be expected to sit still.

Indirect measurements of the soft tissues surrounding the mandible can be obtained using stereophotogrammetry or anthropometry. Although these modalities provide less information about the bony tissue of the mandible itself, they are more useful for routine evaluation of the mandibular size of the infant because they do not involved ionizing radiation and there is no need for sedation. 3D facial measurements of the normal and micrognathic infant are available using 3D surface scanners [9, 10]. A recent study showed good correlation between surface measurements and interior mandibular volume [11].

2D cephalometry or CT can be used to obtain direct information on the bony tissues of the face. MRI does not provide sufficient information on the bony tissue, to give reliable direct measurements of the mandible, though there are now special ‘bone’ sequences available, which improve the assessment of the skull base and vault. [12] However, to the best of our knowledge, there are no papers on mandibular size in infants using MRI. Another reason that makes routine evaluation with MRI difficult in this age group is that sedation is needed to obtain high resolution images of the mandible. Also, measurements of the facial bones have been taken from foetal and neonatal human cadaver’s specimens [13]. Cephalometric analyses of the mandible using 2D lateral cephalograms from 0 to 2 years of age have been documented [4]. However, 2D cephalometry has shown to be less accurate than three-dimensional (3D) measurements on CT using 3D landmarking software [14–18]. CT remains the best imaging modality for measurements of the bony tissue of the face, because of its excellent contrast between soft tissue and bone and ultrafast scan times ruling out the need for sedation. However, imaging methods requiring ionizing radiation, like CT, must be avoided in the infant because of its potential harmful effects in the long term. Therefore, CT at this age is normally restricted as a pre-operative diagnostic and planning tool or for skull trauma. There are several studies performed using 3D CT datasets to evaluate the morphological differences of syndromic skulls compared to normal [19, 20]. To date, to the best of our knowledge, no 3D linear measurements of the mandible in the young infant on CT has been published.

The aim of this cross-sectional study is to provide more insight in the size and growth of the normal mandible in infants using 3D CT. The ultimate goal of this study is to provide growth charts and reference values using linear measurements of 7 landmarks on 3D CT scans in infants aged 0 to 2 years. With this information, a more objective evaluation of micrognathia in the postnatal period is obtained.

## Materials and methods

### Subjects

A multi-centre study was needed to obtain a sufficient number of CT scans. In 2015 CT scans were obtained from 5 hospitals: Sophia Children’s Hospital, Erasmus Medical Centre, Rotterdam, The Netherlands; Wilhelmina Children’s Hospital, Utrecht, The Netherlands; Amsterdam Medical Centre, The Netherlands; Maastricht University Medical Centre, The Netherlands and Boston Children’s Hospital, Boston, USA. We retrospectively assessed all available CT scans in these hospitals between the age of 0 and 2 years old. Scans were included if the mandible was fully visible and when the mandible was not affected by trauma or a disease that could possibly affect mandibular growth. For example, when the patient was scanned for a possible facial trauma, we did not include patients in which the mandible was involved. Scans were also not included when patients were born prematurely or developmental defects were present that could influence mandibular growth, such as Robin sequence. In addition to trauma, we included patients with isolated craniosynostosis, abscesses, unknown soft tissue lesions, swelling of the soft tissues of the face and external ventricular drains. To assess whether isolated craniosynostosis patients can be considered having a normal mandibular size, we performed a one-way ANCOVA analysis with age as a covariate to compare a difference in mandibular size between isolated craniosynostosis compared to non-craniosynostosis scans.

### Landmarks and linear measurements

**Fig. 1:**
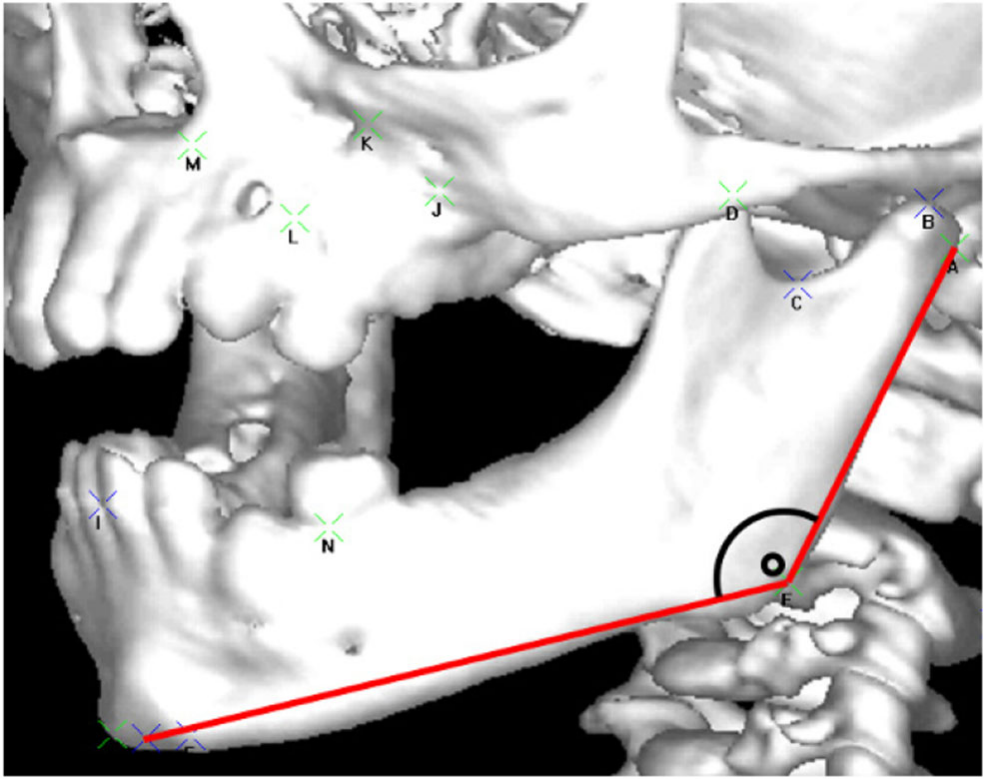
Landmarking in 3D voxel imaging software Robin 3D, the gonial angle inserted as an example

The scans were landmarked using 3D voxel imaging software (Robins 3D, 2013; Robin Richards, London, UK) (Fig. 1). The landmarking process was performed by one person, the first author. The landmark definitions from an earlier study were used [20]. Only 7 landmarks which are essential to the length and angle of the body and ramus of the mandible were used (Table 1). They were landmarked in a horizontal position using the Frankfort horizontal plane.

**Table 1:** Landmarks used for linear measurements of the mandible

	Landmark	Description
CoP (left)	Left condylion posterioris	Most posterior aspect of the left condylar head
Co (left)	Left condylion superioris	Most superior aspect of the left condylar head
Go (left)	Left gonion	Point on the left mandibular angle, defined by dropping a perpendicular line from the intersection point of the tangent lines to the posterior margin of the mandibular vertical ramus and inferior margin of the mandibular body
Me	Menthon	The most inferior point of the mandibular symphysis
CoP (right)	Right condylion posterioris	Most posterior aspect of the right condylar head
Co (right)	Right condylion superioris	Most superior aspect of the right condylar head
Go (right)	Right gonion	Point on the right mandibular angle, defined by dropping a perpendicular line from the intersection point of the tangent lines to the posterior margin of the mandibular vertical ramus and inferior margin of the mandibular body

The Hounsfield units (HU) threshold was set to bone window depending on the ossification of the skull around the value of 255 HU.

Linear measurements were defined as shown in Table 2. The linear distances between the three-dimensional Cartesian coordinates were calculated as the Euclidean distance.

$$ \mathrm{Euclidean}\ \mathrm{distance}\ \mathrm{between}\ {A}_{x,y,z}\ \mathrm{and}\ {B}_{x,y,z}=\sqrt{{\left({A}_x-{B}_x\right)}^2+{\left({A}_y-{B}_y\right)}^2+{\left({A}_z-{B}_z\right)}^2\ } $$

**Table 2:** Linear distances and gonial angle

Linear distance	Description
CoGo (ramus)	Euclidean distance between condylion superioris (Co) and gonion (Go)
GoMn (corpus)	Euclidean distance between gonion (Go) and menthon (Mn)
CoPCoP (bicondylar width)	Euclidean distance between condylion posterioris (CoP) left and condylion posterioris (CoP) right
GoGo (bigonial width)	Euclidean distance between gonion (Go) left and gonion (Go) right
CoMn	Euclidean distance between condylion superioris (Co) and menthon (Mn)
Gonial angle	${\cos}^{-1}\left(\frac{CoGo^2+{GoMn}^2-{CoMn}^2}{2\times CoGo\times GoMn}\right)$

The length of the ramus was defined as the distance between the condylion superioris and the gonion. The length of the body was defined as the distance between the gonion and the menthon. The bigonial width was defined as the distance between the right gonion and the left gonion. The bicondylar width was defined as the distance between the right condylion posterioris and the left condylion posterioris. The gonial angle was defined as the angle between the condylion posterioris-gonion-menthon, as demonstrated in Fig. 1.

### Statistical analysis

To determine the landmark reliability, intra-rater and inter-rater reliability was measured. For the intra-rater reliability, 20 randomly chosen mandibles were landmarked in 2 different sittings by one rater with a minimum of a week between sittings, and intra-class correlation (ICC) values were calculated with a two-way mixed effects model for single measurements and absolute agreement definition. For the inter-rater reliability, 20 randomly chosen mandibles were landmarked by two independent observers, and intra-class correlation (ICC) values were calculated with a two-way random effects model for single measurements and absolute agreement definition.

Polynomial regression was used to construct growth curves for length of the ramus, length of the mandibular body, bicondylar width, bigonial width and gonial angle. Model fit was assessed graphically by inspection of regression curves and residuals. Model fit was adequate in all cases so that confidence bounds could be derived from the models directly without using quantile regression.

SPSS (IBM Corp. Released 2012. IBM SPSS Statistics for Windows, Version 21.0. Armonk, NY: IBM Corp.) was used for all analyses. Statistical analysis was performed by the first author.

## Results

One hundred nine CT scans were included. Initially, in this cohort of 109 cases, we included also 58 CT scans of isolated craniosynostosis patients as one of the most frequent indications for the CT scan. However, ANCOVA showed a significant difference (*p* < 0.05) for a larger size of ramus length and bicondylar width in isolated craniosynostosis patients. A mean difference of 1.5–1.6 mm (left-right) ramus length and 1.7 mm bicondylar width was found. We therefore excluded the isolated craniosynostosis scans, and a total of 51 patients were analysed. The majority of the scans (58.8 %) were made between 0 and 12 months of age, and 41.2% of the scans were made between 12 and 24 months of age. A slight majority of the scans are from male subjects. The main reason for CT evaluation in 51.0% of the patients was possible skull trauma. Table 3 shows the patient characteristics by age, sex, and reason for CT scan.

**Table 3:** Patient characteristics

	Frequency	Percentage
Age		
0–6 months	15	29,4%
6–12 months	15	29,4%
12–18 months	10	19,6%
18–24 months	11	21,6%
Sex		
Male	29	56,9%
Female	22	43,1%
Reason for CT		
Trauma*	26	51,0%
Choanal atresia	5	9,8%
Other reason*	20	39,2%
Total		
	51	100%

*Without mandibular involvement or pathology

Intra-rater and inter-rater reliability were calculated as shown in Table 4. The ICC of the intra-rater reliability was for all distances and angles above 0.9. The ICC of the inter-rater reliability was for all distances above 0.9 and for the gonial angle above 0.8.

**Table 4:** Intra- and inter-rater reliability

Linear measurement	Intra-rater ICC	Inter-rater ICC
Left ramus	0.977	0.977
Left corpus	0.958	0.964
Right ramus	0.979	0.967
Right corpus	0.912	0.978
Bicondylar width	0.974	0.945
Bigonial width	0.991	0.971
Co-Mn L	0.997	0.994
Co-Mn R	0.991	0.996
Gonion angle R	0.958	0.945
Gonion angle L	0.955	0.891

**Fig. 2:**
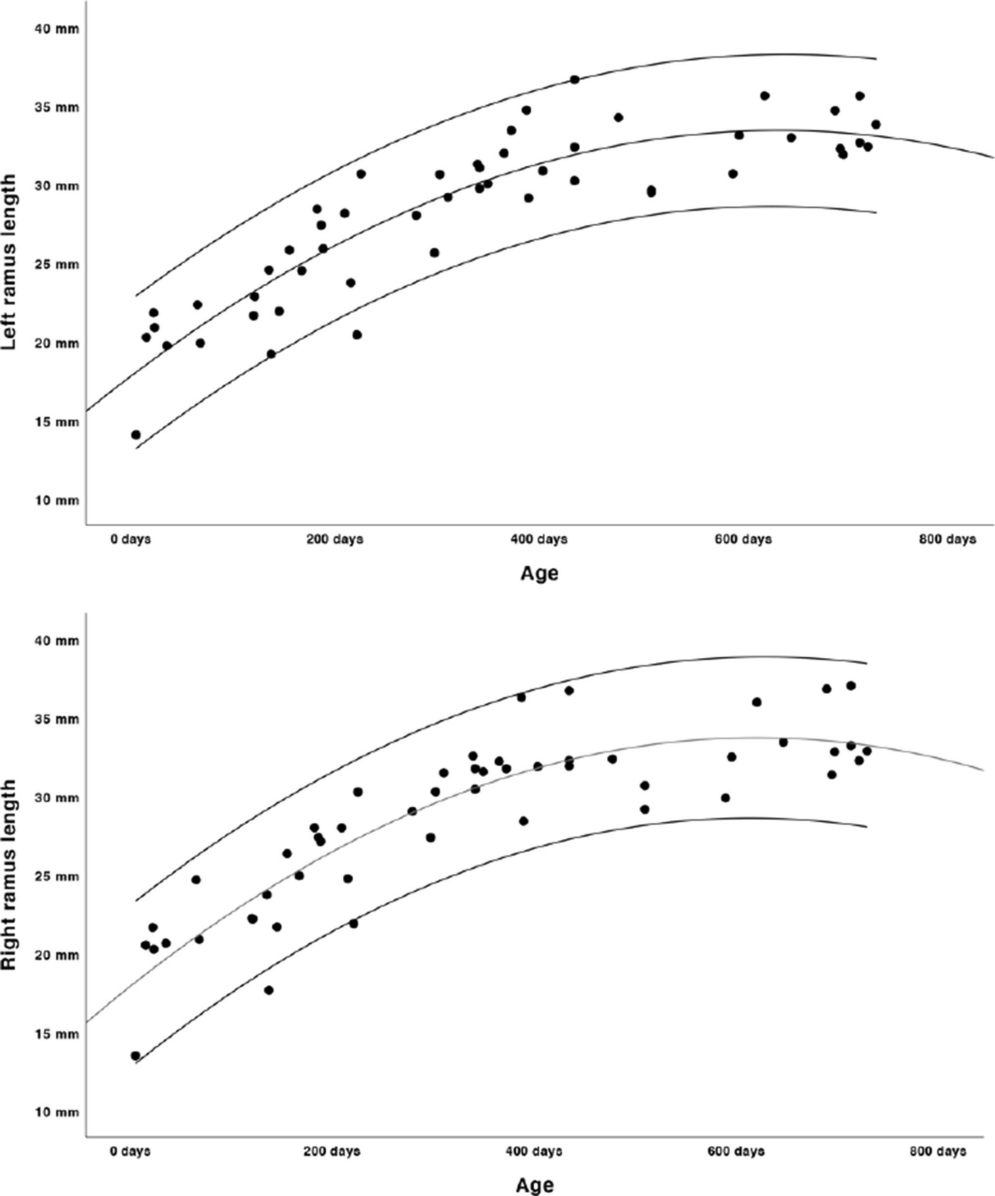
Length of the left and right ramus of the mandible (quadratic growth pattern, length in mm’s, age in days)

**Fig. 3:**
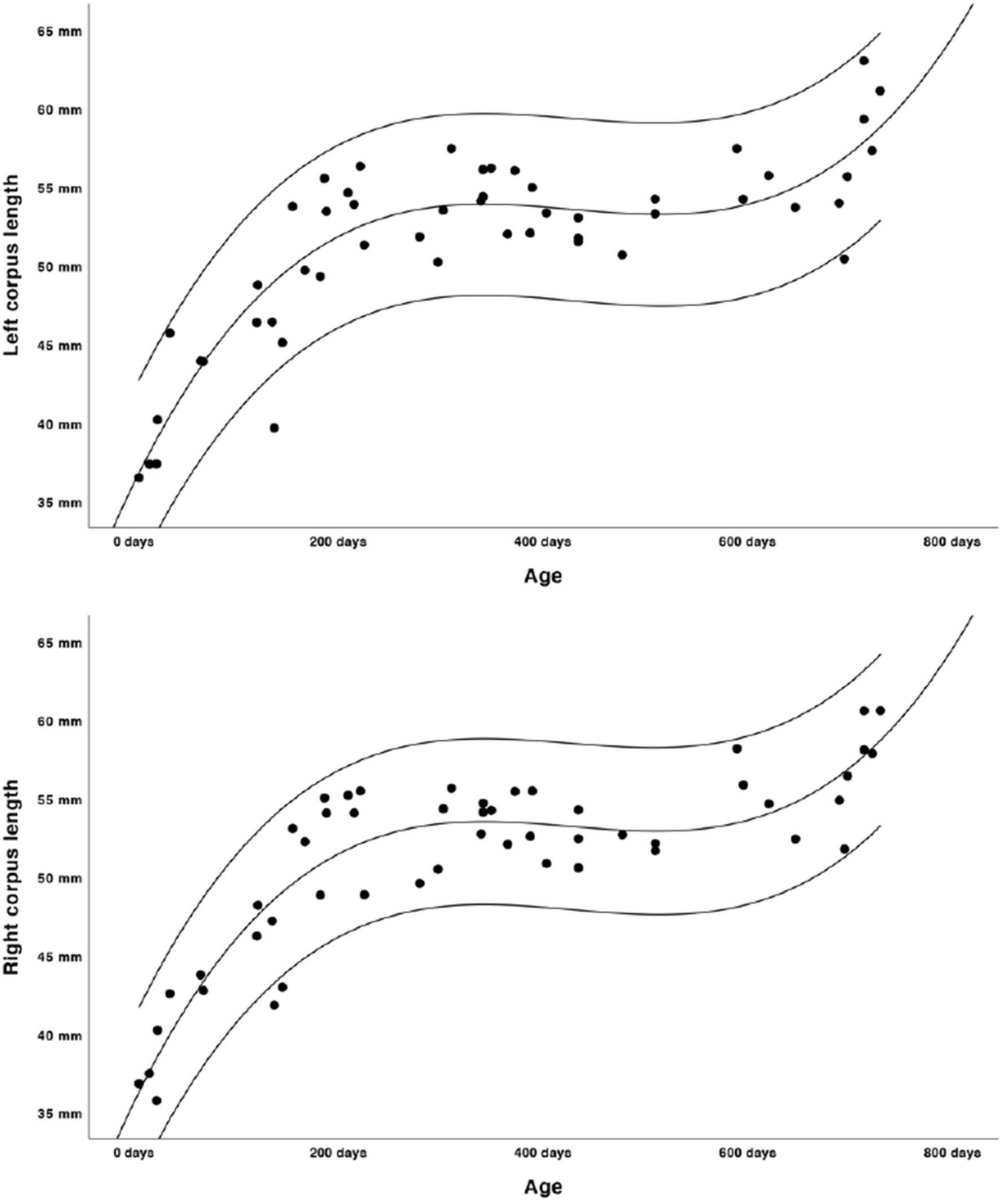
Length of the left and right body of the mandible (cubic growth pattern, length in mm’s, age in days)

**Fig. 4:**
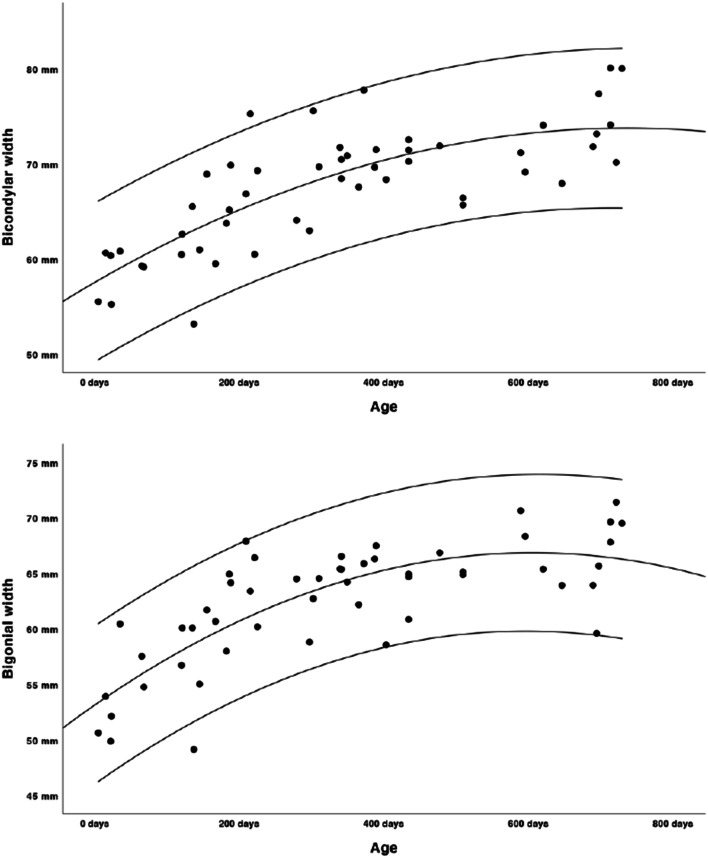
Bicondylar and bigonial width of the mandible (quadratic growth pattern, length in mm’s, age in days)

**Fig. 5:**
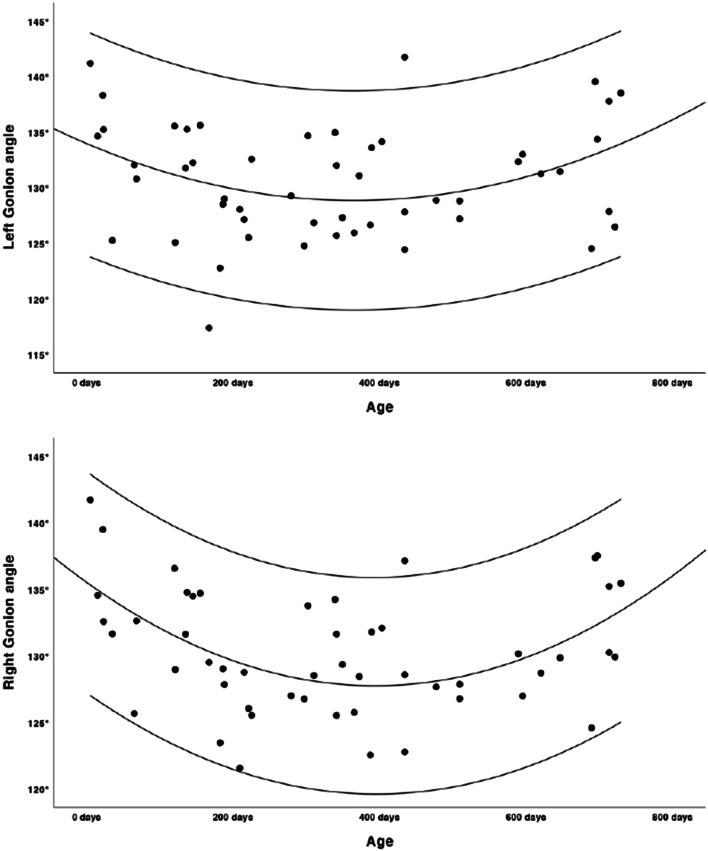
Left and right gonial angle (quadratic growth pattern, angle in degrees, age in days)

**Fig. 6:**
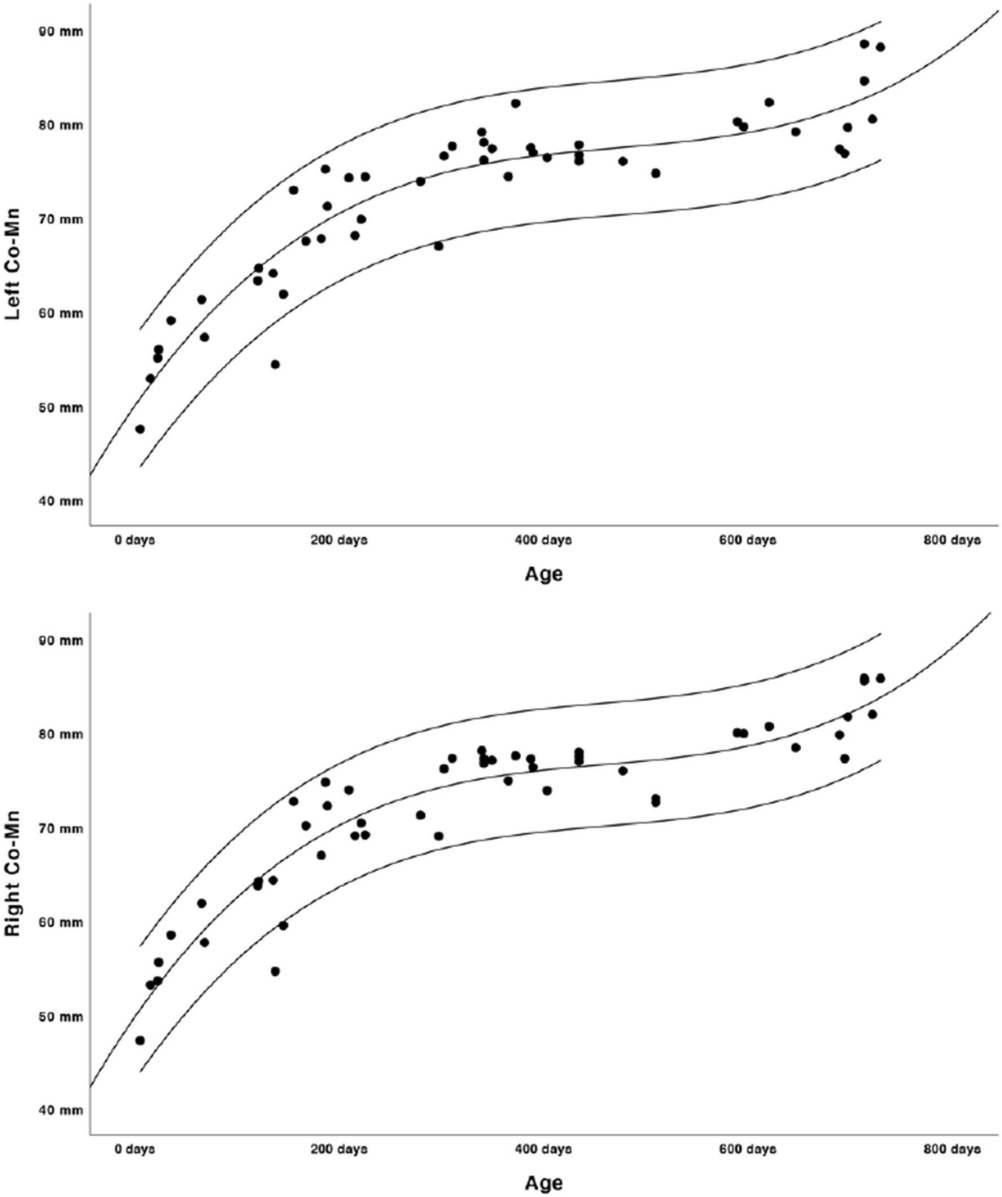
Left and right overall mandibular growth, distance between the condyle and menthon (cubic pattern, length in mm’s, age in days)

Growth charts were modelled as shown in Figs. 2, 3, 4, 5 and 6. Regression lines and individual prediction intervals were calculated, so that the 2.5^th^ and 97.5^th^ percentiles were produced. Ramal height shows a quadratic regression line. There is a decline in the last part of the regression line of the ramal height. The mandibular body length shows a cubic regression line. The bicondylar width and bigonial width show quadratic regression lines. The overall mandibular length (the distance between the condyle and menthon) shows a cubic growth pattern. Figures 2, 3, 4 and 6 show that the slope of the regression lines is steepest in the first 6 months and that the slope decreases in the months thereafter. The gonial angle shows a quadratic regression line. Additional regression results are provided as supplementary table. Descriptive statistics of the Euclidean distances between the landmarks were calculated as shown in Table 5.

**Table 5:** Measurements (length in mm’s; angle in degrees)

	0–6 months (*n*=15)		6–12 months (*n*=15)		12–18 months (*n*=10)		18–24 months (*n*=11)	
	Mean	SD	Mean	SD	Mean	SD	Mean	SD
CoGo (right)	21.9	3.5	29.1	3.0	32.2	2.7	33.5	2.2
CoGo (left)	21.9	3.3	28.3	3.2	32.1	2.6	33.2	1.6
GoMn (right)	44.0	5.3	53.4	2.2	52.8	1.7	56.5	2.9
GoMn (left)	44.3	5.1	54.1	2.1	53.1	1.7	56.5	3.6
GoGo	56.0	4.2	64.1	2.4	64.5	2.8	66.9	3.6
CoPCoP	60.4	4.0	68.5	4.2	70.5	3.4	73.5	4.1
Gonial angle (Right)	132.8	4.8	128.1	3.3	128.6	4.4	131.5	4.3
Gonial angle (left)	131.5	6.4	128.8	3.3	130.4	5.0	132.5	4.9
CoMn (right)	60.3	6.9	73.8	3.4	75.9	2.0	81.5	3.0
CoMn (left)	60.4	6.7	74.2	3.7	76.9	2.1	81.5	4.0

The mandible increases more in size vertically (the mandibular ramus) than horizontally (the mandibular body).

## Discussion

In this study on modelling mandibular growth curves for the first 2 years of life, we found the mandible showed the greatest increase in size in the first 6 months and growth rates decreased thereafter. These growth models provide insight into normal mandibular development in the first 2 years of life. In the graphs, a few outliers can be seen, but the landmarking process showed good reliability. Although the dataset consisted not of truly normal patients as it is not ethical to perform CT scans of healthy young infants, we included patients with various reasons for the scan and excluded scans of patients of which mandibular growth could have been possibly affected. We also excluded isolated craniosynostosis patients, since we found significant differences in ramus length and bicondylar width compared to the other reasons for CT scanning.

Liu et al. showed, using seven longitudinal 2D cephalograms of 48 individuals between birth and 5 years of age, that the greatest growth changes occur in the first 6 months and that growth velocity decreases progressively later in life. Their results demonstrated that overall mandibular length showed the greatest growth changes, followed by ramus height (vertical growth) and then corpus length (horizontal growth). The gonial angle decreased 2.8° and 2° in males and females, respectively. Our findings correspond with their results [4]. Hutchinson et al. performed two studies on cadaveric mandibles of unknown age; standard techniques for age estimation were used. In the first study, they found that the average mandibular body length was 37 mm at 0–11 months (*n*=41) and 47 mm at 12–24 months (*n*=8); the average maximum body length (our Co-Mn measurement) was 48 mm at 0–11 months (*n*=41) and 67 mm at 12–24 months (*n*=8). Our measurements were approximately 10 mm higher than this cadaveric study. In the second study, Hutchinson and colleagues found that the average mandibular body length was 42 mm at 0–12 months (*n*=56) and 52 mm at 12.5–36 months (*n*=17); the average maximum length of the mandible was 56 mm at 0–12 months (*n*=56) and 72 mm at 12.5–36 months (*n*=17); and the average bicondylar width was 62 mm at 0–12 months (*n*=56) and 74 mm at 12.5–36 months (*n*=17). Our measurements were approximately 5 mm higher when compared to this study. Both studies used comparable definitions of the several lengths. As their cohorts consisted of both cadaveric and skeletonized specimens, we think it is possible that shrinkage of the specimens can partially explain why their measurements are smaller than our results [21].

Roelfsema et al. measured the mandibular body length on 3D prenatal ultrasound. They found a mean of 29.8 mm at 34 weeks of pregnancy [22]. Our first postnatal measurements showed values in the range of 35–45 mm. A study of foetuses found a ramus length of 20 mm and a gonial angle of 139° at 39 weeks, which is also comparable to our first postnatal measurements [23].

An earlier study showed that mandibular size is limited in syndromic craniosynostosis. They found that the mandible had a shorter body length, larger ramus height to body length ratio and an obtuse gonial angle compared to age- and sex-matched controls. They found a certain ramus height measurement (Ar-Go) to be larger in size compared to normal, although another measurement of the ramus height we used (Co-Go) was not significantly different. They also found a larger protrusion/retrusion angle, suggesting a protruded mandible [24]. For isolated craniosynostosis to the best of our knowledge, there are no indications that mandibular growth is affected. Although we did not expect that isolated craniosynostosis influenced the results, we performed an ANCOVA analysis to rule this out. Ramus height and bicondylar width showed a significantly larger size. This is an interesting finding as this previous study in syndromic craniosynostosis also found ramus height to be larger [24]. As this was not the primary aim of this study, we cannot draw any conclusions from these results. Further research has to be done to evaluate if mandibular growth is affected in isolated craniosynostosis.

There are a few limitations in our study. We found a declining regression line of the ramus length, bicondylar width and bigonial width in the last part of the second year of life. As we can assume that there is no decrease in size, this could be explained by the fact that there is not a sufficient number of scans in the last part of the second life year as seen in Table 3. We do not expect this last part of the regression line to be a true representation of growth, as growth could possibly slow down but size will not decline in the first 2 years of life. The gonial angle shows great variability between the age of 0 and 2 years, which is an indication that the mandibular shape varies greatly in our cohort. We do not believe this quadratic regression line is an indication that the gonial angle decreases and later increases in life, but that it rather decreases slightly in the first 2 years. Because there were no longitudinal CT data available, this data is cross-sectional and does not provide growth information on an individual level. Although we did a multi-centre study and searched all available CT scans in the several hospitals, the power of this study is still low due to the few useable CT scans. Because of the paucity of scans, we have not been able to make separate analyses based on patient characteristics (e.g. race or gender), although it has been shown that these factors may influence the size and growth of the mandible [4, 25]. Although we have evaluated the clinical information to exclude pathology that could influence the size of the mandible, patients underwent CT scans for reasons as skull trauma or choanal atresia. This is not a ‘normal’ population, although we believe that these reasons for the scan do not influence the results. Despite these limitations, we were able to model mandibular growth curves which provide insight into mandibular growth patterns in the first 2 years of life.

We analysed the data as to whether quantile regression should be preferred over linear regression. As the plotted figures showed that our data was normally distributed, linear regression was chosen as analysis for the growth curve analysis.

More studies gathering and analysing scans, preferably longitudinally, from patients with these anomalies at young age are needed to understand the growth pattern of the hypoplastic mandible in syndromic patients. Mandibular hypoplasia is seen in several anomalies, such as isolated and syndromal Robin sequence, craniofacial microsomia, Treacher Collins and Nager syndrome [26]. In a recent review, it was shown that the scientific evidence for the concept of catch-up growth, as often quoted in the literature on Robin sequence, is weak [27].

As stated, CT scans need to be avoided for routine diagnostics in healthy children because of ionizing radiation. When a CT scan is performed in children with severe mandibular developmental abnormalities, our models could assist in quantifying the deviation from normal mandibular growth. When longitudinal scans would be available, our models could also provide insight whether growth is normalizing.

In summary, our modelled growth models of the mandible from 0 to 2 years of age demonstrate that it increased more in size vertically (the mandibular ramus) than horizontally (the mandibular body). Most of the mandibular growth occurs in the first 6 months and growth rates decreased thereafter. Future studies are mandatory to provide information on postnatal normal and congenitally hypoplastic mandibular development. Ideally, methods should be developed that do not use ionizing radiation, such as three-dimensional surface stereophotogrammetry.

## Supplementary information

ESM 1 (DOCX 16 kb)
