# Supplementary material for: Modelling growth curves of the normal infant’s mandible: 3D measurements using computed tomography
Source: Clin Oral Investig. 2021 Apr 16;25(11):6365–75. doi: 10.1007/s00784-021-03937-1 (PMC8531114; doi:10.1007/s00784-021-03937-1)
Supplement: Supplementary file 1 — (DOCX 16 kb) [file 784_2021_3937_MOESM1_ESM.docx]

**Supplementary Table – Regression results**

|  | B (95% CI) | P-value | R^2^ |
| --- | --- | --- | --- |
| **Left ramus length** | Intercept = 17,81 (16,01 – 19,61) Age = 0,05 (0,038 - 0,061) Age^2^ = -3,89E-5 (0,00 – 0,00) | Intercept: p<0,001  Age: p<0,001  Age^2^: p<0,001 | 0,815 |
| **Right ramus length** | Intercept = 17,92 (16,01 – 19,83) Age = 0,051 (0,039 – 0,063) Age^2^ = -4,11E-5 (0,00 – 0,00) | Intercept: p<0,001  Age: p<0,001  Age^2^: p<0,001 | 0,798 |
| **Left body length** | Intercept = 36,15 (33,33 – 38,96) Age = 0,133 (0,099 – 0,168) Age^2^ = 0,00 (0,00 – 0,00) Age^3^ =2,54E-7 (0,00 – 0,00) | Intercept: p<0,001  Age: p<0,001  Age^2^: p<0,001  Age^3^: p<0,001 | 0,785 |
| **Right body length** | Intercept = 35,64 (33,07 – 38,21) Age = 0,135 (0,103 – 0,166) Age^2^ = 0,00 (0,00 - 0,00) Age^3^ = 2,57E-7 (0,00 – 0,00) | Intercept: p<0,001  Age: p<0,001  Age^2^: p<0,001  Age^3^: p<0,001 | 0,818 |
| **Bicondylar width** | Intercept = 57,54 (54,45 – 60,63) Age = 0,044 (0,025 – 0,063) Age^2^ = -2,99E-5 (0,00 – 0,00) | Intercept: p<0,001  Age: p<0,001  Age^2^: p = 0,02 | 0,615 |
| **Bigonial width** | Intercept = 53,13 (50,47 – 55,77) Age = 0,045 (0,029 – 0,062) Age^2^ = -3,744E-5 (0,00 – 0,00) | Intercept: p<0,001  Age: p<0,001  Age^2^: p = 0,001 | 0,609 |
| **Left gonial angle** | Intercept = 133,97 (130,24 – 137,71) Age = -0,028 (-0,052 - -0,005) Age^2^ = 3,84E-5 (-0,00 – 0,00) | Intercept: p<0,001  Age: p= 0,020  Age^2^: p = 0,014 | 0,121 |
| **Right gonial angle** | Intercept = 135,52 (132,44 – 138,61) Age = -0,039 (-0,059 - -0,020) Age^2^ = 5,02E-5 (0,00 – 0,00) | Intercept: p<0,001  Age: p<0,001  Age^2^: p<0,001 | 0,260 |
| **Left overall mandibular length** | Intercept = 50,036 (46,56 – 53,51) Age = 0,155 (0,112 – 0,198) Age^2^ = 0,00 (0,00 – 0,00) Age^3^ = 2,17E-7 (0,00 – 0,00) | Intercept: p<0,001  Age: p<0,001  Age^2^: p<0,001  Age^3^: p = 0,001 | 0,866 |
| **Right overall mandibular length** | Intercept = 49,83 (46,66 – 53,01) Age = 0,157 (0,118 – 0,196) Age^2^ = 0,00 (0,00 – 0,00) Age^3^ = 2,34E-7 (0,00 – 0,00) | Intercept: p<0,001  Age: p<0,001  Age^2^: p<0,001  Age^3^: p<0,001 | 0,885 |
